# SOURCE: a phase 3, multicentre, randomized, double-blind, placebo-controlled, parallel group trial to evaluate the efficacy and safety of tezepelumab in reducing oral corticosteroid use in adults with oral corticosteroid dependent asthma

**DOI:** 10.1186/s12931-020-01503-z

**Published:** 2020-10-13

**Authors:** Michael E. Wechsler, Gene Colice, Janet M. Griffiths, Gun Almqvist, Tor Skärby, Teresa Piechowiak, Primal Kaur, Karin Bowen, Åsa Hellqvist, May Mo, Esther Garcia Gil

**Affiliations:** 1 National Jewish Health, Denver, CO USA; 2 Respiratory & Immunology, BioPharmaceuticals R&D, AstraZeneca, Gaithersburg, MD USA; 3 Translational Science and Experimental Medicine, Research and Early Development, Respiratory & Immunology, BioPharmaceuticals R&D, AstraZeneca, Gaithersburg, MD USA; 4 Late Respiratory & Immunology, BioPharmaceuticals R&D, AstraZeneca, Gothenburg, Sweden; 5 Development Operations, BioPharmaceuticals R&D, AstraZeneca, Mississauga, ON Canada; 6 Amgen, Thousand Oaks, CA USA; 7 Biometrics, Late Respiratory & Immunology, BioPharmaceuticals R&D, Gaithersburg, MD USA; 8 Biometrics, Late Respiratory & Immunology, BioPharmaceuticals R&D, Gothenburg, Sweden; 9 Global Medical Respiratory, BioPharmaceuticals R&D, AstraZeneca, Barcelona, Spain

**Keywords:** Alarmin, Asthma, Asthma control, Exacerbation, Epithelial, Oral corticosteroids, SOURCE, Steroid-sparing, Tezepelumab, TSLP

## Abstract

**Background:**

Many patients with severe asthma continue to experience asthma symptoms and exacerbations despite standard-of-care treatment. A substantial proportion of these patients require long-term treatment with oral corticosteroids (OCS), often at high doses, which are associated with considerable multiorgan adverse effects, including metabolic disorders, osteoporosis and adrenal insufficiency. Tezepelumab is a human monoclonal antibody that blocks the activity of the epithelial cytokine thymic stromal lymphopoietin. In the PATHWAY phase 2b study (NCT02054130), tezepelumab significantly reduced exacerbations by up to 71% in adults with severe, uncontrolled asthma. Several ongoing phase 3 trials (SOURCE, NCT03406078; NAVIGATOR, NCT03347279; DESTINATION, NCT03706079) are assessing the efficacy and safety of tezepelumab in patients with severe, uncontrolled asthma. Here, we describe the design and objectives of SOURCE, a phase 3 OCS-sparing study.

**Methods:**

SOURCE is an ongoing phase 3, multicentre, randomized, double-blind, placebo-controlled study to evaluate the effect of tezepelumab 210 mg administered subcutaneously every 4 weeks on OCS dose reduction in adults with OCS-dependent asthma. The study comprises a 2-week screening and enrolment period, followed by an OCS optimization phase of up to 8 weeks and a 48-week treatment period, which consists of a 4-week induction phase, followed by a 36-week OCS reduction phase and an 8-week maintenance phase. The primary objective is to assess the effect of tezepelumab compared with placebo in reducing the prescribed OCS maintenance dose. The key secondary objective is to assess the effect of tezepelumab on asthma exacerbation rates. Other secondary objectives include the proportion of patients with a reduction in OCS dose (100% or 50% reduction or those receiving < 5 mg) and the effect of tezepelumab on lung function and patient-reported outcomes.

**Conclusions:**

SOURCE is evaluating the OCS-sparing potential of tezepelumab in patients with OCS-dependent asthma. SOURCE also aims to demonstrate that treatment with tezepelumab in patients with severe asthma is associated with reductions in exacerbation rates and improvements in lung function, asthma control and health-related quality of life, while reducing OCS dose.

**Trial registration:**

NCT03406078 (ClinicalTrials.gov). Registered 23 January 2018. https://clinicaltrials.gov/ct2/show/NCT03406078

## Background

Oral corticosteroids (OCS) are widely used for the maintenance treatment of severe asthma and asthma exacerbations in patients who cannot achieve symptomatic control when treated with inhaled corticosteroids (ICS) and long-acting β_2_ agonists (LABAs) [1]. Approximately 20–60% of patients with severe or uncontrolled asthma require long-term treatment with OCS as an additional asthma controller medication [2]. Although OCS have been shown to be effective in controlling airway inflammation in asthma, chronic use (≥ 6 months) is associated with potentially debilitating side effects including, but not limited to, metabolic disorders (e.g. diabetes) [3, 4], osteoporosis [5], adrenal insufficiency [3], pneumonia [6], cardiovascular adverse events, cataracts and weight gain [7]. OCS-related complications are dose-dependently associated with long-term (≥ 6 months) OCS use at daily doses as low as 5 mg or less of prednisolone (or an equivalent dose of another corticosteroid) [8]. Long-term exposure to systemic corticosteroids is associated with increased costs and healthcare resource utilization in patients with severe asthma [2]. Therefore, asthma treatments that allow a reduction in OCS dose or complete cessation of treatment with OCS, while maintaining control of asthma symptoms, would reduce morbidity and improve patient health-related quality of life (HRQoL). Current biologic treatments (e.g. benralizumab, dupilumab and mepolizumab) may be viable alternative therapies to OCS, and can assist in tapering and discontinuing OCS therapy [9–11]. These treatments precisely target factors contributing to asthma pathogenesis and severity, such as inflammation-promoting cytokines and interleukins [9, 10, 12], and are indicated for the treatment of patients with specific severe asthma phenotypes (e.g. eosinophilic or allergic asthma) [13–15]. Targeting an upstream mediator of inflammatory pathways may have a broader effect on airway inflammation, and may provide more effective asthma control in a broader patient population than currently available biologic treatments.

Thymic stromal lymphopoietin (TSLP) is an epithelial-derived cytokine (also termed an ‘alarmin’) implicated in the initiation and persistence of airway inflammation, and is a key upstream regulator of many inflammatory pathways in asthma [16–18]. Expression of TSLP is increased in the airways of patients with asthma and correlates with disease severity [19, 20]. TSLP is released in response to triggers associated with asthma exacerbations, such as allergens and viruses [21, 22], and has been reported to be a potential mediator of corticosteroid resistance in patients with severe asthma [23]. Therefore, blocking TSLP in patients with OCS-dependent asthma may potentially reduce TSLP-mediated corticosteroid insensitivity, permitting reductions in or cessation of OCS treatment.

**Fig. 1:**
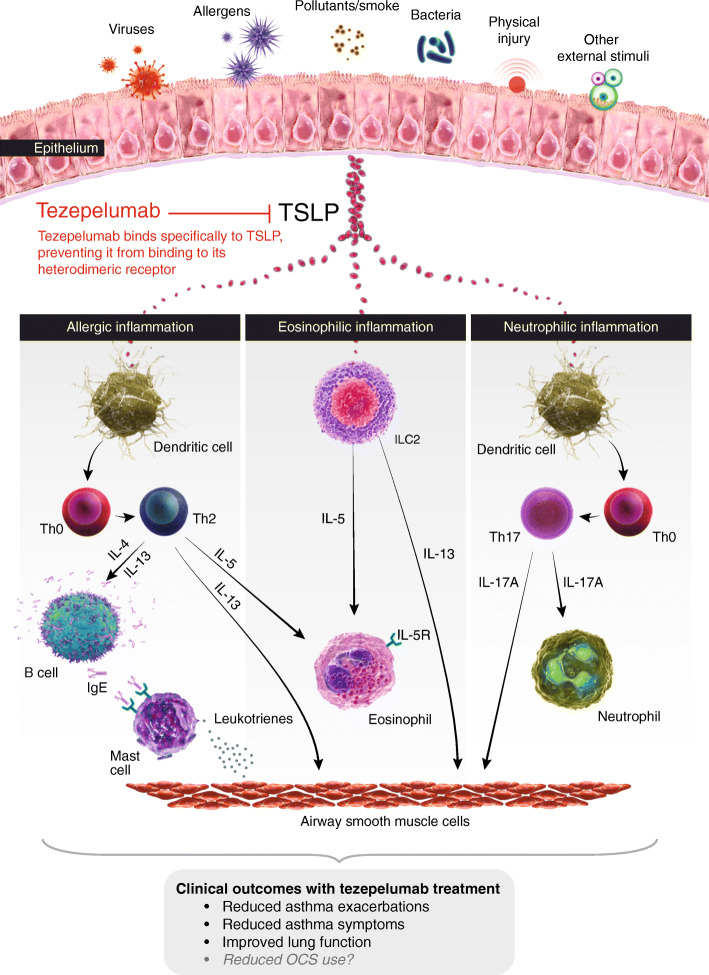
Mechanism of action by which tezepelumab improves clinical outcomes in patients with asthma. TSLP is released from the airway epithelium in response to insults such as viruses, allergens and pollutants, triggering an inflammatory cascade. Overexpression of TSLP can result in pathologic inflammation that can lead to asthma exacerbations, symptoms, and physiological effects such as bronchoconstriction and airway hyperresponsiveness and remodelling. Tezepelumab specifically blocks TSLP from binding to its heterodimeric receptor, thereby inhibiting the production of various inflammatory cytokines and cell types. Treatment with tezepelumab has thus far been shown to reduce blood eosinophil count, IgE, IL-5, IL-13and FeNO. The effects of tezepelumab on OCS use will be investigated in SOURCE. *FeNO*, fractional exhaled nitric oxide; *IgE*, immunoglobulin E; *IL*, interleukin; *ILC2*, type 2 innate lymphoid cell; *OCS,* oral corticosteroid; *Th*, T-helper; *TSLP*, thymic stromal lymphopoietin

Tezepelumab is a human monoclonal antibody (immunoglobulin G2λ) that binds specifically to TSLP, blocking it from interacting with its heterodimeric receptor (Fig. 1) [24, 25]. In a proof-of-concept study, inhibition of TSLP with tezepelumab reduced early and late bronchoconstriction after inhaled allergen challenge in adults with mild, atopic asthma [25]. In the phase 2b PATHWAY study (ClinicalTrials.gov identifier: NCT02054130), tezepelumab significantly reduced asthma exacerbations over 52 weeks by up to 71% compared with placebo in patients with severe, uncontrolled asthma, irrespective of baseline biomarker status [24, 26], and improved lung function, asthma control and patient HRQoL [24]. In addition, tezepelumab reduced exacerbations in a subset of patients from PATHWAY who were receiving maintenance treatment (for ≥ 6 months) with OCS [24]. The efficacy and safety of tezepelumab in patients with severe, uncontrolled asthma are being investigated in a number of ongoing phase 3 trials (including SOURCE, ClinicalTrials.gov identifier: NCT03406078; NAVIGATOR, ClinicalTrials.gov identifier: NCT03347279; and DESTINATION, ClinicalTrials.gov identifier: NCT03706079). In addition, the effect of tezepelumab on airway inflammation is being assessed in the ongoing phase 2 CASCADE study (ClinicalTrials.gov identifier: NCT03688074). This article describes the design and objectives of the phase 3 SOURCE study, which aims to demonstrate that tezepelumab enables a reduction in OCS use in adults with OCS-dependent asthma.

## Methods

### Study design

SOURCE is an ongoing phase 3, multicentre, randomized, double-blind, placebo-controlled, parallel-group study that aims to evaluate the effect of tezepelumab 210 mg administered subcutaneously every 4 weeks (Q4W) on OCS dose reduction in adults with OCS-dependent asthma. Patient recruitment for SOURCE has been completed. A total of 150 patients have been randomized from 47 study sites in 7 countries globally. Patients were stratified by region (Central and Eastern Europe, Western Europe and North America, and rest of the world). Eligible patients must have been receiving OCS for the treatment of asthma for at least 6 months before screening, and must have been taking a stable dose of 7.5–30 mg (prednisone or prednisolone) daily or daily equivalent for at least 1 month before screening. Patients must also have been receiving medium- or high-dose ICS for 12 months before screening. Patients who were receiving medium-dose ICS, must have had their ICS dose increased to a high dose for at least 3 months before screening. In addition, patients must have been receiving a LABA with or without additional controller medications, for at least 3 months before screening. Patients who were using additional maintenance asthma controller medications, according to standard-of-care practice, were permitted to enter the study if use of these medications had been documented for at least 3 months before screening. Patients must also have had at least one asthma exacerbation in the 12 months before screening. Patients who had received any marketed or investigational biologic were permitted to enter the study, provided the appropriate washout period was fulfilled (4 months or 5 half-lives, whichever was longer, before visit 1). Key additional inclusion and exclusion criteria are shown in Table 1.

**Table 1:** Key inclusion and exclusion criteria

**Key inclusion criteria**	
• Men or women, 18–80 years old, weight ≥ 40 kg at visit 1 • Documented physician-diagnosed asthma for ≥ 12 months before visit 1, and receiving medium- or high-dose ICS (as per GINA 2017 guidelines [1]) for 12 months before visit 1 • Documented physician-prescribed LABA and high-dose ICS (total daily dose corresponding to fluticasone propionate > 500 μg dry powder formulation equivalent) for ≥ 3 months before visit 1 • Additional maintenance asthma controller medications (e.g. LAMA, LTRA, theophylline, secondary ICS and cromones) are permitted if documented for ≥ 3 months before visit 1 • Received OCS for the treatment of asthma for ≥ 6 months before visit 1 and receiving a stable dose of prednisone or prednisolone 7.5–30 mg daily or daily equivalent for ≥ 1 month before visit 1 • Morning pre-bronchodilator FEV_1_ < 80% predicted at either visit 1 or visit 2 • Documented historical FEV_1_ reversibility of ≥ 12% and ≥ 200 mL (15–30 min after administration of four puffs of albuterol/salbutamol) in the 12 months before visit 1 or at visit 1 or visit 2 • History of ≥ 1 asthma exacerbation event ≤ 12 months before visit 1 • Received optimized OCS dose for ≥ 2 weeks before randomization	
**Key exclusion criteria**	
• Any clinically important pulmonary disease, other than asthma, associated with high peripheral eosinophil counts • Any disorder that could, in the opinion of the investigator, affect the safety of the patient or influence study findings • Any clinically significant infection requiring antibiotic or antiviral treatment in the 2 weeks before visit 1 or during the enrolment period • Helminth or parasitic infection diagnosed in the 6 months before visit 1 that has not been treated with, or is unresponsive to, standard-of-care therapy • History of cancer, HIV, or hepatitis B or C • Current smokers or patients with a smoking history of ≥ 10 pack-years • History of chronic alcohol or drug abuse ≤ 12 months before visit 1 • Tuberculosis requiring treatment ≤ 12 months before visit 1 • Use of any marked or investigational biologic agent in the 4 months or 5 half-lives before visit 1, or any investigational non-biologic agent in the 30 days or 5 half-lives before visit 1 • Use of any immunosuppressive medication in the 12 weeks before randomization • History of anaphylaxis after biologic therapy • Pregnant, breastfeeding or lactating • If, during the optimization period, asthma control requires an OCS dose < 7.5 mg or > 30 mg and/or if asthma control is still maintained after three consecutive OCS dose reductions	

*FEV*_*1*_ Forced expiratory volume in 1 s, *GINA* Global initiative for asthma, *HIV* Human immunodeficiency virus, *ICS* Inhaled corticosteroid, *LABA* Long-acting β_2_ agonist, *LAMA* Long-acting muscarinic antagonist, *LTRA* Leukotriene receptor antagonist, *OCS* Oral corticosteroid

The total study population was monitored to ensure that approximately 35% of patients had a blood eosinophil count of at least 300 cells/μL at enrolment. No other restrictions on blood eosinophil counts were imposed.

**Fig. 2:**
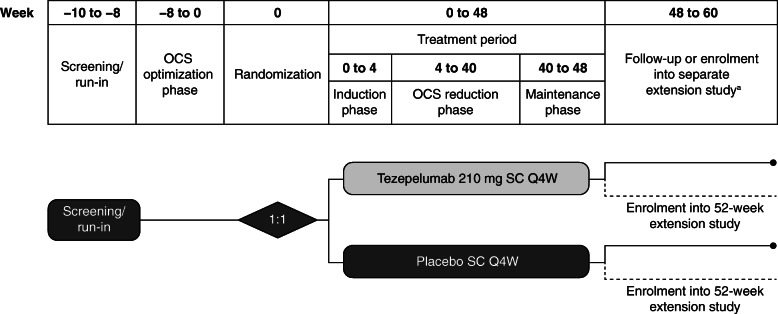
Study design. ^a^Patients who enrol in the extension study on the same day as the end-of-treatment visit in SOURCE will not attend follow-up visits at week 54 and week 60. *OCS*, oral corticosteroids; *Q4W*, every 4 weeks; *s.c.*, subcutaneous

The study comprises a 2-week screening and enrolment period, followed by an OCS optimization phase of up to 8 weeks and a 48-week treatment period, which consists of a 4-week induction phase, followed by a 36-week OCS reduction phase and an 8-week maintenance phase (Fig. 2).

During the optimization phase, the optimized OCS doses (the minimum doses at which asthma control is maintained) will be determined for all patients. Asthma control was defined according to the criteria in Table 2. OCS reduction can occur every 2 weeks. In the dosing interval of 7.5 mg to 10 mg dose reductions will be 2.5 mg and in the dosing interval of > 10 mg to 30 mg dose reductions will be 5.0 mg. Once the optimized OCS dose is reached, no further OCS dose reductions will be performed during this phase. During the optimization phase, patients whose asthma is controlled with an OCS dose of less than 7.5 mg, or whose asthma control is maintained after three consecutive OCS dose reductions, will be screen failed. The optimized OCS dose will be kept stable for 2 weeks before randomization, and will be considered the baseline OCS dose at start of treatment with tezepelumab or placebo. This dose will also be maintained throughout the 4-week induction phase after the first dose of study treatment (tezepelumab 210 mg or placebo).

**Table 2:** Asthma control criteria required for OCS dose reduction

Criteria	Definition of asthma control
1	Morning PEF ≥ 80% of mean^a^ morning measures compared with mean baseline^b^ measures
2^c^	Increase of ≤ 2 nights with asthma-related awakenings (requiring rescue medication) over a 7-day period compared with baseline^b^
3	Mean^a^ SABA rescue medication use ≤ 4 puffs/day above the baseline^b^ mean and < 12 puffs/day on all days in the previous 14 days
4	No asthma exacerbation requiring increased systemic corticosteroids or hospitalization since the previous visit
5	Investigator judges the patient’s asthma control to be sufficient to allow OCS dose reduction
6	No signs/symptoms of adrenal insufficiency (at OCS dose reductions < 5 mg)

^a^Mean values considered for each down-titration visit will be from electronic diary records completed for 14 days before the visit

^b^Baseline values for the optimization phase will be calculated from electronic diary records completed for ≥ 10 out of 14 days before visit 2. Similarly, baseline values for the reduction phase will be calculated from ≥ 10 out of 14 days of data collected before the randomization visit

^c^The number of nights with asthma-related awakenings requiring rescue medication will be counted from the most recent 7 days of available data

*OCS* Oral corticosteroid, *PEF* Peak expiratory flow, *SABA* Short-acting β_2_ agonist

During the OCS reduction phase of the treatment period, daily OCS doses greater than 10 mg will be reduced by 5 mg every 4 weeks (Table 3). Doses of 10 mg/day or less will be reduced by 2.5 mg every 4 weeks. Each patient who is eligible for OCS reduction will follow the same tapering or reduction schedule. After 4 weeks at a dose of 2.5 mg/day, a further reduction to 0 mg/day will be considered. However, if, in the judgement of the investigator, OCS dose reduction should be more gradually tapered, dose reductions of 1–1.25 mg/day every 1–4 weeks will be considered. Patients who do not meet the asthma control criteria for continued down-titration (Table 2) will return to the previous effective dose (i.e. the higher dose level before not meeting the asthma control criteria for down-titration) or the investigator may decide to keep the patient on the OCS dose at which the down-titration criteria were not met. In patients who do not meet the asthma control criteria for continued down-titration, the investigator may consider further attempts at dose reduction during subsequent visits.

**Table 3:** OCS dose titration schedule during the reduction phase

	OCS dose per day, mg								
Optimized dose at week 0	Week								
	4	8	12	16	20	24	28	32	36
30.0	25.0	20.0	15.0	10.0	7.5	5.0	2.5	0.0	0.0
25.0	20.0	15.0	10.0	7.5	5.0	2.5	0.0	0.0	0.0
20.0	15.0	10.0	7.5	5.0	2.5	0.0	0.0	0.0	0.0
15.0	10.0	7.5	5.0	2.5	0.0	0.0	0.0	0.0	0.0
12.5	7.5	5.0	2.5	0.0	0.0	0.0	0.0	0.0	0.0
10.0	7.5	5.0	2.5	0.0	0.0	0.0	0.0	0.0	0.0
7.5	5.0	2.5	0.0	0.0	0.0	0.0	0.0	0.0	0.0

*OCS* Oral corticosteroid

The maintenance phase will assess maintenance of asthma control, while patients continue to take the OCS dose achieved at the end of the reduction phase. Patients who experience an exacerbation during this period (after the OCS bolus/burst is completed) will either return to a higher OCS dose than the dose they were receiving, or maintain the same OCS dose throughout the maintenance phase. Patients will be followed up for 12 weeks post-treatment.

Patients were randomized 1:1 to receive subcutaneous tezepelumab 210 mg Q4W (administered using a single-use vial and syringe) or placebo Q4W until week 44. Neither treatment will be as administered at week 48. Patients who complete the treatment period as defined in the study protocol (week 48) are eligible to enrol in a separate extension study (DESTINATION); these patients will not attend follow-up visits at weeks 54 and 60.

Owing to the COVID-19 virus pandemic, the SOURCE protocol was amended. This amendment was necessary to address the issue of social distancing and the possibility that site visits would be limited. If site visits by patients were not possible, some efficacy and safety data may not have been collected. In addition, patients would not have been able to receive study drug. The amendment specifically allowed for virtual visits (instead of site visits) to collect appropriate efficacy and safety information, and for at-home dosing of tezepelumab or placebo when possible.

Written, informed consent was obtained from all patients before enrolment into the study. The study is being conducted in accordance with the principles established in the Declaration of Helsinki and the International Conference on Harmonisation guidelines for good clinical practice.

### Objectives and outcome measures

A summary of the primary and secondary objectives and endpoints for this study is given in Table 4. The primary objective is to assess the effect of tezepelumab compared with placebo in reducing the prescribed daily OCS maintenance dose. This will be assessed by calculating the percentage reduction from baseline to week 48 in the prescribed daily OCS maintenance dose while not losing asthma control. The key secondary objective is to evaluate the effect of tezepelumab compared with placebo on asthma exacerbations, which will be assessed by the annualized asthma exacerbation rate (AAER). Additional, supportive assessments related to exacerbations include time to first asthma exacerbation, rate of asthma exacerbations associated with emergency room or urgent care visits or hospitalizations, and the proportion of patients without any asthma exacerbations.

**Table 4:** Primary and secondary objectives and endpoints

Objective	Endpoint
*Primary objective*	
Assess the effect of tezepelumab compared with placebo in reducing the prescribed OCS maintenance dose in adults with severe, uncontrolled asthma	Percentage reduction from baseline in daily OCS dose at week 48, defined as: 1. 90–100% reduction 2. 75–< 90% reduction 3. 50–< 75% reduction 4. 0–< 50% reduction 5. no change or any increase
*Key secondary objective*	
Assess the effect of tezepelumab on asthma exacerbations compared with placebo	AAER • Time to first asthma exacerbation • Rate of asthma exacerbations associated with ER visits, urgent care visits or hospitalization • Proportion of subjects who did not experience an asthma exacerbation
*Other secondary objectives*	
Assess the effect of tezepelumab on the prescribed OCS daily maintenance dose	Proportion of patients with 100% reduction from baseline in daily OCS dose at week 48 Proportion of patients with daily OCS dose ≤ 5 mg at week 48 Proportion of patients with ≥ 50% reduction from baseline in daily OCS dose at week 48
Assess the effect of tezepelumab on pulmonary function compared with placebo	Change from baseline in pre-BD FEV_1_
Assess the effect of tezepelumab on asthma symptoms and other asthma control metrics, compared with placebo	Change from baseline in: • weekly mean daily ASD score • weekly mean rescue medication use • weekly mean morning and evening PEF • weekly mean number of night-time awakenings • ACQ-6 score
Assess the effect of tezepelumab on asthma-related and general health-related quality of life compared with placebo	Change from baseline in: • AQLQ(S)+12 total score • EQ-5D-5L score
Assess the effect of tezepelumab on healthcare resource use and productivity loss owing to asthma	Asthma-specific resource use (e.g. unscheduled physician visits, use of other asthma medications) WPAI + CIQ scores
Assess the effect of tezepelumab on biomarkers	Change from baseline in FeNO, peripheral blood eosinophil count
Evaluate the pharmacokinetics and immunogenicity of tezepelumab	Pharmacokinetics: serum trough concentrations Immunogenicity: incidence of ADAs

*AAER* Annualized asthma exacerbation rate, *ACQ-6* Asthma Control Questionnaire-6 items, *ADA* anti-drug antibody, *AQLQ(S)+12* Asthma Quality of Life Questionnaire standardized for patients 12 years and older, *ASD* Asthma Symptom Diary, *BD* Bronchodilator, *EQ-5D-5L* 5-dimension 5-level EuroQol questionnaire, *ER* Emergency room, *FeNO* Fractional exhaled nitric oxide, *FEV*_*1*_ forced expiratory volume in 1 s, *OCS* Oral corticosteroids, *PEF* Peak expiratory flow, *WPAI + CIQ* Work Productivity and Activity Impairment Questionnaire and Classroom Impairment Questionnaire

Additional secondary objectives related to OCS use include the effect of tezepelumab compared with placebo on the prescribed OCS daily maintenance dose, as assessed by the proportions of patients with 100% reduction and at least a 50% reduction in OCS dose at week 48, and by the proportion of patients with a daily OCS dose of no more than 5 mg at week 48. Further secondary objectives include the effect of tezepelumab on lung function (pre-bronchodilator forced expiratory volume in 1 s) and inflammatory biomarkers (including fractional exhaled nitric oxide, blood eosinophils and total serum immunoglobulin E). Inflammatory biomarkers will not be measured during the optimization phase and will be measured at selected visits during the reduction phase and the follow-up period; results will be blinded for the study sites and sponsor. Healthcare resource use and patient-reported outcomes will be assessed, including asthma control (using the Asthma Control Questionnaire-6), asthma symptoms (using the Asthma Quality of Life Questionnaire standardized for patients 12 years and older [AQLQ(S)+12]) and HRQoL.

SOURCE will investigate a number of exploratory outcomes, including but not limited to: the effect of tezepelumab on post-bronchodilator lung function; the effect of tezepelumab on patient health status (using St George’s Respiratory Questionnaire [SGRQ]); the effect of tezepelumab on circulating biomarkers, including markers of type 2 inflammation; the relationship between baseline inflammatory biomarkers and the effect of tezepelumab on OCS dose reduction and clinical efficacy; and the daily average exposure to systemic corticosteroids (including temporary increase in systemic corticosteroid treatment for worsening of asthma symptoms and exacerbations). Some exploratory endpoints will also be evaluated in subgroups according to high and low baseline blood eosinophil counts.

Throughout the study, serum samples will be collected for the analysis of tezepelumab pharmacokinetics (serum concentrations) and analysis of the potential immunogenicity of tezepelumab, measured by the incidence of anti-drug antibodies and characterization of their neutralizing potential. The safety and tolerability of tezepelumab will be evaluated throughout the study by monitoring adverse events, serious adverse events, vital signs, clinical chemistry, haematology and urinalysis parameters, and digital electrocardiograms. A data safety monitoring board (an expert advisory group that will function independently of all other individuals associated with the study) will evaluate cumulative safety and other clinical trial data at regular intervals, and make appropriate recommendations based on available data. The effect of tezepelumab on glucocorticoid toxicity will be assessed using the glucocorticoid toxicity index (GTI) [27]. The GTI is a composite measure that captures common glucocorticoid toxicities that are sensitive to differing cumulative glucocorticoid doses over time. Individual items within the GTI are weighted relative to each other for severity, and the GTI can measure changes from baseline in glucocorticoid toxicity.

### Statistical considerations

Statistical analyses of efficacy outcomes will be performed on all patients who receive at least one dose of tezepelumab or placebo, according to randomized treatment assignment (full analysis set), and statistical analyses of safety and anti-drug antibody incidence will be performed according to treatment received. Pharmacokinetic analyses will be performed on all patients in the full analysis set who were randomized to receive tezepelumab; samples assumed to be affected by factors such as certain important protocol deviations will not be included (e.g. use of non-permitted medications or receipt of incorrect study medication).

For the primary endpoint (the percentage reduction from baseline in daily OCS dose while not losing asthma control at week 48), percentage change from baseline is defined as ([final OCS dose – baseline OCS dose])/baseline OCS dose)*100, where the final dose is the prescribed asthma maintenance OCS dose at visit 18 (week 48) or the dose received when asthma stability was last verified, expressed as dose per day (full details of derivations for specific situations are detailed in the Statistical Analyses Plan, signed off prior to the study unblinding). Percentage reductions from baseline will be categorized as: 1) 90–100%; 2) 75–< 90%; 3) 50–< 75%; 4) 0–< 50%; or 5) no reduction or any increase. The primary endpoint will be analysed using a proportional odds (ordinal logistic regression) model, with the ordered percentage reduction category number (1–5) at week 48 as the response variable. Treatment and region will be included as factors in the model. Baseline OCS dose will be included as a continuous (linear) covariate in the model. Patients who discontinue treatment early will be encouraged to undergo all study-related visits and procedures for the 48-week study period; consequently. In patients who withdraw from the study and are lost to follow-up, the final OCS dose will be defined as one dose level higher than the dose received when asthma stability was last verified (i.e. no change in OCS dose for ≥ 2 weeks). If asthma stability was not verified, the final OCS dose will be the baseline dose. Derivations for patients impacted by COVID-19 will be documented in the Statistical Analysis Plan.

The actual percentage reductions in daily OCS dose at week 48 will also be summarized descriptively and compared between treatments using a Wilcoxon rank sum test, stratified by region (van Elteren test). Binary (responder) endpoints that support the primary objective with regards to OCS dose reduction will be summarized using frequency tables. The odds ratio (tezepelumab:placebo) and 95% confidence interval will be estimated for each endpoint from a logistic regression model with factors for treatment and region, and baseline OCS dose included as a continuous (linear) covariate. Other binary endpoints will be summarized and analysed similarly.

AAER over 48 weeks (the key secondary endpoint) will be analysed using a negative binomial regression model, with the total number of asthma exacerbations experienced by a patient over the 48-week treatment period as a response variable. Treatment, region and history of exacerbations (≤ 2 or > 2 in the previous 12 months) will be included as covariates in the model. The logarithm of the time at risk for exacerbation during the study (excludes duration of an exacerbation and the 7-day period after an exacerbation, during which patients are not considered at risk of exacerbation) will be used as an offset variable in the model.

Changes from baseline in continuous variables for other endpoints will generally be analysed using a mixed model for repeated measures, which will estimate the effect of treatment at week 48 and the 95% confidence interval for each endpoint. The response variable in the model will be the change from baseline at each scheduled post-randomization visit up to and including week 48, irrespective of whether the patient remained on treatment and/or received other treatments. Treatment, visit, region and treatment-by-visit interaction will be included as factors in this model. The baseline value of the corresponding endpoint will also be included in the model as a continuous linear covariate.

The overall type 1 error rate will be strongly controlled at the 0.05 level across the primary and key secondary endpoints. To account for multiplicity, a hierarchical testing strategy will be used to test the primary endpoint and then the key secondary endpoint. With approximately 76 patients per treatment group, assuming an odds ratio of 2.75 and the proportional odds assumption, and that the proportion of patients in each dose reduction category is similar to what was observed in the mepolizumab OCS-sparing study [9], it is estimated that, using the levels of error control described, the power for the primary endpoint will be at least 90% [9]. For the key secondary endpoint (AAER over 48 weeks), assuming a placebo rate of 1.3 exacerbations per year in this study population, a conservative assumption on the dispersion parameter and uniform dropout of 10%, there will be more than 80% power to reject the null hypothesis for rate ratios up to 0.39, using a two-sided 5% significance level.

Additional statistical analyses required due to COVID-19 will be pre-specified in the statistical analyses plan before sponsor unblinding and will be described when the results for this study are reported.

## Discussion

Regular use of OCS is often required to treat patients with severe asthma that remains uncontrolled despite treatment with ICS and other controller medications; however, chronic OCS use is associated with potentially debilitating adverse outcomes [1, 3–5]. Therefore, a treatment that allows a reduction in OCS dose or cessation of OCS treatment, while maintaining asthma control, would greatly improve HRQoL for patients with severe, OCS-dependent asthma.

A recent study in human blood and airway innate lymphoid cells from patients with asthma has suggested that TSLP is a mediator of corticosteroid resistance [23]. Blocking TSLP with tezepelumab has been shown to be an effective strategy for reducing exacerbations and improving lung function and asthma control in patients with severe, uncontrolled asthma in the phase 2b PATHWAY study [24]. Therefore, blocking TSLP in patients with OCS-dependent asthma may potentially reverse corticosteroid insensitivity caused by TSLP, permitting reductions in OCS dose or cessation of OCS treatment. Furthermore, in the PATHWAY study, tezepelumab reduced exacerbations irrespective of baseline blood eosinophil counts and levels of other inflammatory biomarkers, suggesting that it may be suitable for the treatment of a broad population of patients with different asthma phenotypes [24]. Based on the findings from PATHWAY, tezepelumab was granted Breakthrough Therapy Designation by the US Food and Drug Administration in 2019 for patients with severe asthma without an eosinophilic phenotype, who are receiving ICS/LABA with or without OCS and additional asthma controllers [28]. The OCS-sparing potential of tezepelumab in patients with different asthma phenotypes will be investigated in SOURCE, which has enrolled patients with high and low baseline blood eosinophil counts (66% with a blood eosinophil count < 300 cells/μL). The study population was monitored to ensure that a sufficient number of patients with both high (≥ 300 cells/μL) and low (< 300 cells/μL) blood eosinophil counts were enrolled, in order to adequately understand the effect of tezepelumab in a broad population of patients with OCS-dependent asthma. In a previous study of biologic treatment for asthma, which did not have restrictions on minimum requirements for blood eosinophil counts at baseline, the proportion of patients with high blood eosinophil counts (42%) was comparable with that for SOURCE [11].

This study has a similar design and endpoints to previous placebo-controlled, OCS-sparing studies of mepolizumab [9], benralizumab [10] and dupilumab [11]; however, its duration (48 weeks) is longer than that of previous studies (24–40 weeks). The OCS reduction period in previous studies of mepolizumab and benralizumab (16–20 weeks) did not prove long enough to allow patients receiving an OCS dose above 25 mg/day and 12.5 mg/day, respectively, to reduce their OCS dose to 0 mg [9, 10]. The longer OCS reduction phase in SOURCE (36 weeks) will provide patients with at least one additional opportunity to attempt to reduce their OCS dose if they lose asthma control. OCS dose reductions will occur at 2-week and 4-week intervals during the optimization phase and reduction phase, respectively, which is similar to previous OCS-sparing studies of mepolizumab [9], benralizumab [10] and tralokinumab [12]. In addition, the maintenance phase is longer in SOURCE (8 weeks) than in previous studies of mepolizumab and benralizumab (4 weeks) [9, 10]. This will allow more time to assess the efficacy of tezepelumab in reducing the maintenance OCS dose.

Additional secondary endpoints (e.g. exacerbation rate, lung function, asthma control and HRQoL) and exploratory endpoints (e.g. biomarkers of response to treatment) will be included in SOURCE based on greater understanding of the potential effects of tezepelumab following the phase 2b PATHWAY study. SOURCE will assess patient quality of life using SGRQ in addition to the AQLQ(S)+12, which is in contrast with previous OCS-sparing studies that have only used one of these measures [9–11, 29]. The GTI can objectively measure changes in the side effects of corticosteroid therapy over time, and will provide a better understanding of the impact of the OCS-sparing effects of tezepelumab treatment. Patients enrolled in SOURCE will have the option to enrol in a double-blind, placebo-controlled, safety extension study (DESTINATION, ClinicalTrials.gov identifier: NCT03706079) for an additional year.

## Conclusions

Patients with severe, uncontrolled asthma who are receiving long-term treatment with OCS represent a population with a significant unmet need for new treatments that obviate the requirement for maintenance OCS therapy, and that provide greater improvements in asthma outcomes than standard-of-care therapies and current biologics. SOURCE is evaluating the effect of tezepelumab in reducing OCS use in patients with OCS-dependent asthma, irrespective of blood eosinophil count, and aims to demonstrate its OCS-sparing potential and impact on glucocorticoid toxicity. SOURCE also aims to provide further evidence of findings from earlier clinical studies with tezepelumab, and to demonstrate its potential to provide patients with reductions in exacerbations and improvements in lung function, asthma control and HRQoL, while reducing OCS treatment.

## Data Availability

Not applicable.

## Abbreviations

AAER: Annualized asthma exacerbation rate
ACQ: Asthma control questionnaire
ADA: Anti-drug antibody
AQLQ(S) + 12: Asthma quality of life questionnaire standardized for patients 12 years and older
ASD: Asthma symptom diary
FeNO: Fractional exhaled nitric oxide
FEV_1_: Forced expiratory volume in 1 s
GTI: Glucocorticoid toxicity index
HRQoL: Health-related quality of life
ICS: Inhaled corticosteroids
LABA: Long-acting β_2_ agonist
OCS: Oral corticosteroids
Q4W: Every 4 weeks
SGRQ: St George’s respiratory questionnaire
TSLP: Thymic stromal lymphopoietin
